# Identifying the best combination of vegetation and paving materials to improve the outdoor thermal comfort in a campus courtyard

**DOI:** 10.1038/s41598-025-05742-2

**Published:** 2025-07-01

**Authors:** Zihao Ye, Jing Liu, Zhihuan Huang

**Affiliations:** College of Architecture and Design, University of South China, Hengyang, 421001 China

**Keywords:** ENVI-met, Thermal comfort, Vegetation, Pavement material, Physiological equivalent temperature (PET), Climate-change mitigation, Environmental health, Sustainability, Urban ecology

## Abstract

The urban heat island effect poses significant challenges to urban residents by diminishing thermal comfort and altering outdoor behaviors in hot environments. Although improvements in green space and adjustments to paving materials can alleviate these impacts, their combined effects remain insufficiently studied. This study employed ENVI-met software to simulate nine vegetation-paving material combinations and assess their influence on the thermal environment and thermal comfort of a campus courtyard during summer. Regression analyses were conducted to explore the interactions among these strategies. Air temperature (*T*_*a*_), surface temperature (*T*_*s*_), relative humidity (RH) and physiological equivalent temperature (PET) were used as key indicators to evaluate the effectiveness of each scenario. Results showed that “trees + lawn” and “trees + shrubs + lawn” were most effective in reducing *T*_*a*_, *T*_*s*_ and PET, while increasing RH. In contrast, the “lawn + shrubs” configuration demonstrated limited effectiveness. Among the paving materials, high-reflectivity concrete yielded the greatest reductions in *T*_*a*_ and *T*_*s*_, followed by standard concrete, with asphalt being the least effective. However, high-reflective concrete also led to an increase in PET due to increased radiant heat exposure. These findings provide a valuable foundation for informing microclimate-responsive landscape strategies in campus courtyards under summer conditions.

## Introduction

Urban warming driven by global climate change and the urban heat island effect threatens the thermal safety and comfort of urban residents, as moderate and extreme heat exposure are well-documented contributors to morbidity and mortality^1,2^. According the data from IPCC (2021), the global average surface temperature between 2001 and 2020 was 0.99 °C higher than the preindustrial average temperature^3^. Extreme heat events are expected to occur more frequently and with greater intensity in 21th century^4,5^. Urban green space is widely recognized as one of the most significant Nature-based solutions for mitigating urban warming due to its considerable cost-effective cooling capacity^6^. Previous studies have demonstrated that urban green space can intercept solar radiation and reduce both surface and air temperatures through evapotranspiration and shade provision. Some studies report that parks and green areas can reduce air temperatures by approximately 1 °C, with measurable cooling effects extending up to 250 m into the surrounding urban areas^7,8^. It is more likely that people will use and appreciate the outdoor environments such as parks, squares and streets when they offer a thermal comfortable and healthy environment^9^. Therefore, thermal comfort must be considered in all aspects of urban green spaces to be utilized and enjoyed by residents^10^.

Due to the complexity of the composition of urban green spaces, predictive modeling has become essential for evaluating how climate change may affect their thermal comfort^11^. Among available tools, the ENVI-met model is widely regarded as one of the most effective for simulating the spatial distribution of thermal parameters across diverse climatic contexts and urban morphologies, including variations in building configurations and vegetation types^12,13^. ENVI-met has demonstrated strong performance in numerous studies assessing the thermal exchange processes between vegetation, buildings, and the ground surface under different scenarios. For instance, it has been employed to simulate the thermal benefits of trees^14,15^, evaluate the cooling effects of different green roofs^16^, and analyze the thermal environment in specific urban spaces such as parks^17^ and school courtyards^18^. Moreover, its application in community-scale thermal comfort studies^9,19^ highlights its value in shaping public space design and renewal.

According to data from the National Bureau of Statistics, urban green areas in China have increased by nearly 50% between 2012 and 2021^20^. With the rapid expansion of higher education in China, campus green spaces have become essential large public areas for university students. These spaces typically include playgrounds, lawns, squares and courtyards, often featuring paved surfaces for accessibility. Studies have demonstrated that campus courtyards provide a pleasurable space for students to release frustrations, reduce stress levels, and improve physical and psychological health^21,22^. Given that thermal comfort plays a critical role in influencing students’ willingness to engage in activities within these courtyards^23^, it is necessary to assess and improve their thermal comfort conditions.

In this study, ENVI-met was used to simulate the summer microclimate and field survey was conducted to clarify the validity and applicability of the simulation in a campus courtyard in Hengyang City. This study has three objectives: (1) To assess the thermal comfort of the campus courtyard; (2) To identify the impact factors that affect the thermal comfort; (3) Find out the optimized thermal comfort combination of vegetation and pavement in the courtyard. Air temperature (*T*_*a*_), surface temperature (*T*_*s*_), relative humidity (RH), and physiological equivalent temperature (PET) were used to evaluate the effectiveness of various scenarios in improving thermal comfort and optimizing the thermal environment of campus courtyards during summer. Addressing these issues could provide valuable data for optimizing the microclimate design of campus courtyards in summer.

## Methodology

### Study site

**Fig. 1:**
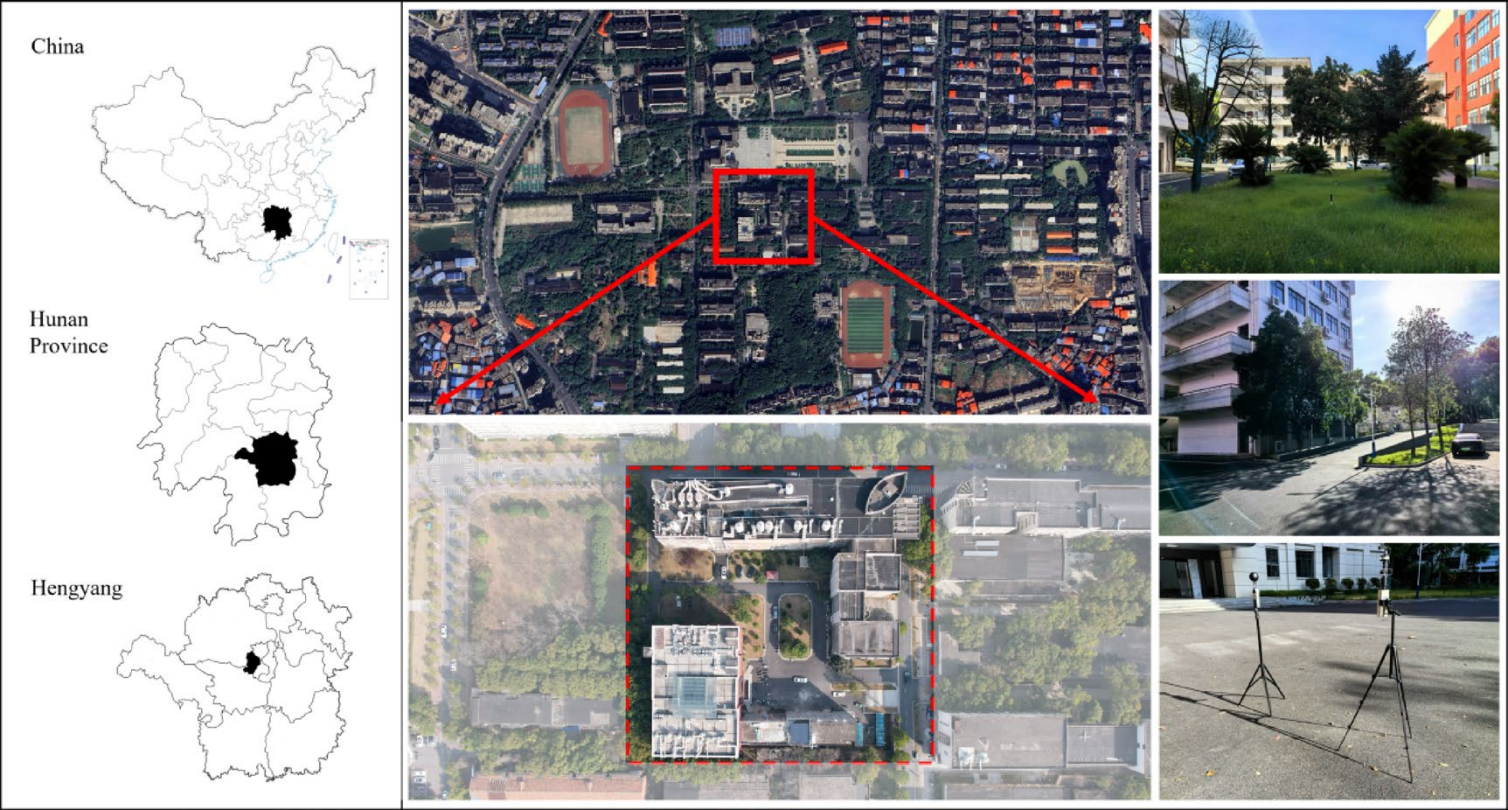
Current situation of the study site.The satellite imagery was obtained from MAP WORLD (www.tianditu.gov.cn).

The study area is located in Hengyang, southern China, where the Xiangjiang River divides the city into two parts. It has a subtropical humid monsoon climate with an annual average temperature of 18 °C. July is the hottest month, characterized by an average high temperature of 35 °C accompanied by high relative humidity. The study site is a courtyard situated within a university campus in the center of Hengyang City, adjacent to Pinghu Park. It features well-developed transportation infrastructure, abundant greenery and several historic buildings. The site spans approximately 0.72 hectares and is bordered by educational and administrative facilities. The tallest building within the site reaches nine stories. Vegetation is abundant, predominantly composed of trees and shrubs (Fig. 1).

### Methods

**Fig. 2:**
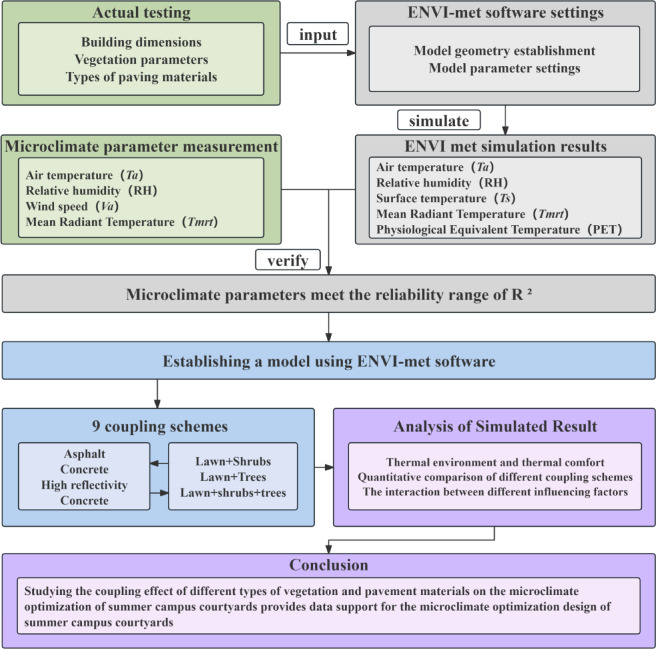
The proposed methodology in this study.

**Fig. 3:**
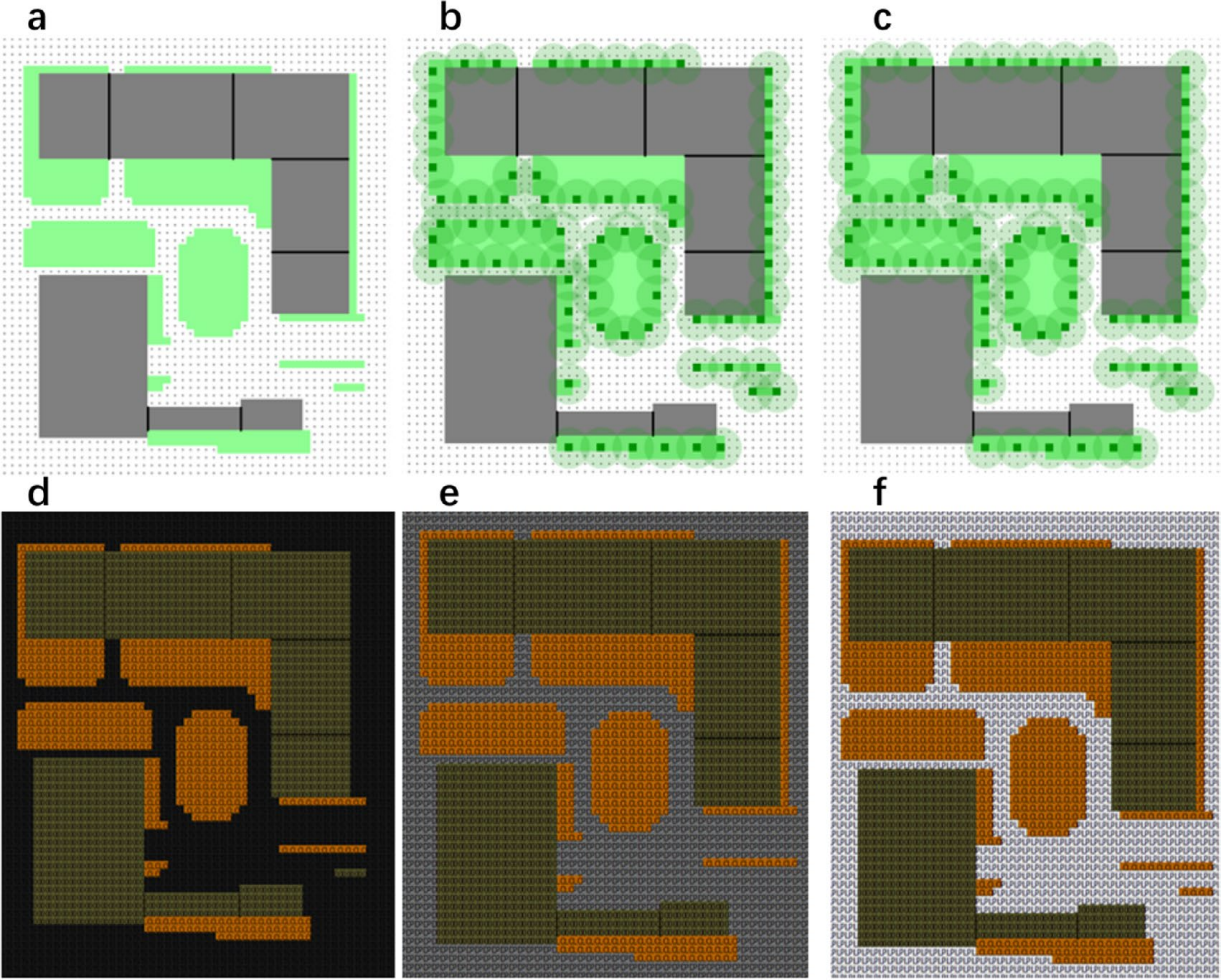
Different types of surface paving and green spaces. **a** Pure lawn; **b** combination of lawn and trees; **c** combination of lawn, trees and shrubs; **d** asphalt paving; **e** concrete paving; **f** high albedo concrete.

The methodological approach of this study consisted of four parts. First, field measurements were conducted to collect microclimate data within the university campus courtyard. Second, an ENVI-met model was developed, and its simulation accuracy was validated against observed data. Third, multiple improvement scenarios combining various landscape elements and paving materials were incorporated into the model to simulate their effects on microclimatic conditions and generate spatial thermal environment maps. Fourth, simulation results were analyzed to quantify the impacts of different vegetation and paving materials on the courtyard’s thermal environment (Figs. 2, 3).

### ENVI-met modeling and parameterization

The ENVI-met Space module, integrated with field survey data, was employed to construct a microclimate model of the study site. Model parameters were derived from historical climate data for Hengyang. Given the relatively small scale of the study area, five nested computational grids were added along the model’s outer boundaries to reduce the influence of external environmental conditions^24^. The model was developed using base data consisting of AutoCAD vector files and high-resolution satellite imagery acquired through ArcGIS. The site image was converted into BMP format and imported into ENVI-met for simulation setup. Data processing and visualization were performed using Microsoft Excel and Origin.

#### Model quantification

**Fig. 4:**
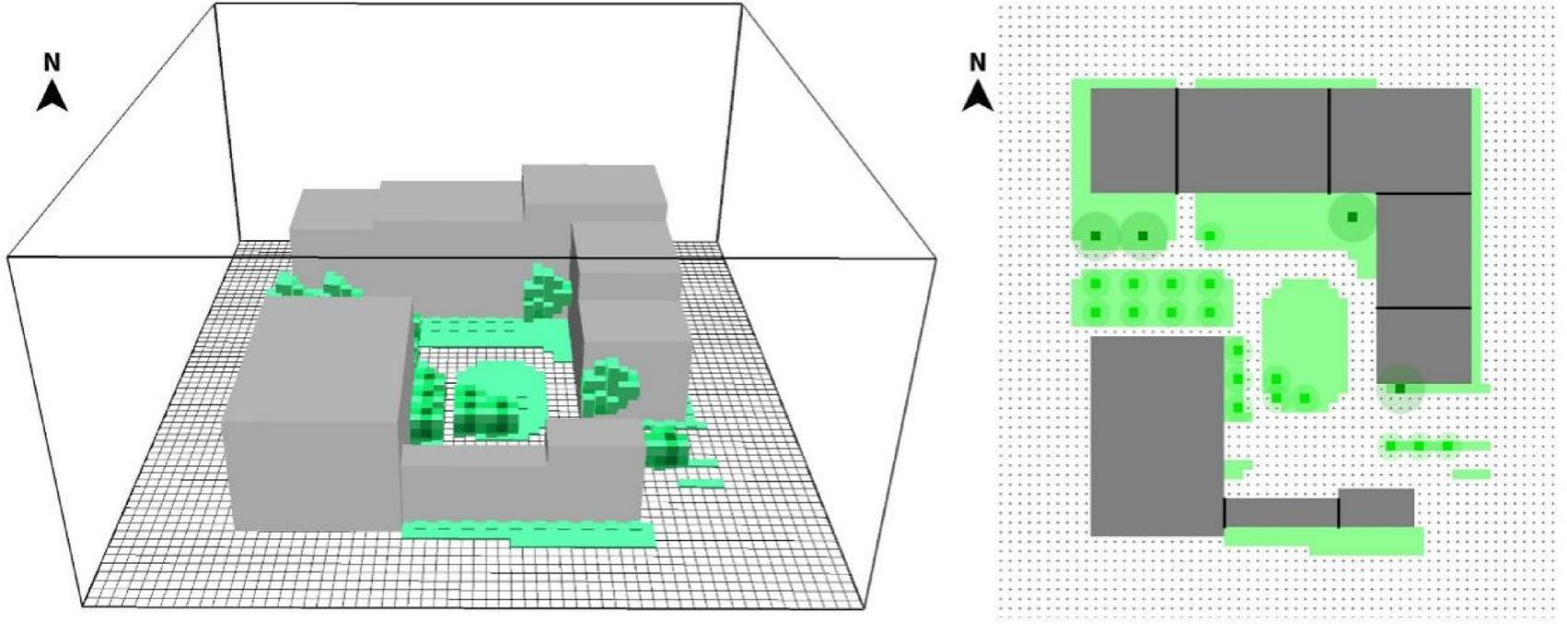
ENVI-met model for studying the current state of the region.

The study employed ENVI-met (version 5.6.1) to model an approximately rectangular study area with dimensions of 82 m × 93 m. To improve computational accuracy, a grid of 60 × 65 cells with a 2 m resolution was applied^25^. Building footprints, vegetation, and surface types were delineated using drone aerial imagery, supplemented by building data from the university’s topographic maps and CAD drawings. The tallest building in the area reaches 27 m. To ensure simulation accuracy, the model’s vertical extent was set to more than twice this height, with 30 grid layers vertically at 2 m resolution. The modeling configuration is illustrated in Fig. 4.

#### Parameter settings

Accurate geographic and climatic data were essential inputs for the ENVI-met simulation. The model was configured with coordinates 26º53′38″ N, 112º34′19″ E, situated in the UTC + 8 time zone (Beijing time) at an elevation of 143 m. Initial boundary conditions were established based on field measurements recorded on July 13, 2024. A detailed summary of these parameters is provided in Table 1. The simulation period spanned from 07:00 to 18:00, with outputs generated at 30-min intervals, covering peak student activity times and focusing on optimizing summer thermal comfort.

**Table 1:** Input parameters used for ENVI-met model configuration.

Existing configuration	Settings
Total area	83 m × 93 m
Model area height	60 m
Buildings	Two contiguous buildings, with a maximum height of 30 m and a minimum height of 12 m
Boundary condition	Full forcing
Wall material	Buildings: Default wall and brick wall
Roof material	Default wall, moderate insulation
Vegetation	Tree: 15.62 m, distinct crown layer Leaf type: deciduous, LAD = 1 m^2^/m^3^
	Shrubs: 1 m, Leaf type: deciduous, LAD = 0.5 m^2^/m^3^
	Grass: 25 cm, LAD = 0.5 m^2^/m^3^
Soil and pavement	Pavements: [0000PG] Concrete pavement, [0000PL] Concrete pavement and [0200ST] Asphalt Road
	Soil: [000000] Default sandy Loam
Soil conditions	Upper layer (0–20 cm): 65% soil humidity, 20 °C initial temperature
	Middle layer (20–50 cm): 70% soil humidity, 20 °C initial temperature
	Deep layer (50–200 cm): 75% soil humidity, 19 °C initial temperature
Roughness height	0.5

Soil conditions	Settings
Simulated date	July 13, 2024
Simulated time period	7: 00–18: 00
Number of grids	60 × 65 × 30
Grid resolution	dx = 2.0 m; dy = 2.0 m; dz = 2.0 m
Wind speed	2.38–4.20 m/s
Wind direction	183.38°–188.64°
Temperature	29.25–32.56 °C
Relative humidity	45.88–58.44%

### Model validation

#### Microclimate measurements

To validate the ENVI-met simulation outputs, air temperature and relative humidity data generated by the model were compared with field meteorological measurements. Both simulations and field experiments were conducted from 07:00 to 18:00 on July 13–15, 2024. These dates were selected due to persistent clear weather and mean daily temperatures exceeding 35 °C, representing typical extreme heat conditions and corresponding to the historically hottest period of summer in the study region^26^. This approach ensured that the simulations captured the most thermally stressful conditions commonly encountered in the campus environment. Microclimate measurements were taken at two locations. The primary measurement point, situated in an open courtyard within the study area, reflected the representative microclimatic conditions and was used for validating the simulation results. The second measurement point, positioned on the rooftop of a nearby building, recorded air temperature (*T*_*a*_), relative humidity (RH), and wind speed (*V*_*a*_) to provide boundary conditions for the ENVI-met model. Environmental data were collected using a portable weather station (PLC-16025) at a height of 1.4 m. The station recorded *T*_*a*_, RH, and *V*_*a*_ at 15-min intervals. Additionally, a 75 mm black globe thermometer (AZ8778) was installed to measure globe temperature (*T*_*ɡ*_). Mean radiant temperature (*T*_*mrt*_) was subsequently calculated using the following equation:1

Equation (1), used to calculate *T*_*mrt*_^27^ and is based on the ISO 7726 standard (1998)^28^, which is widely applied in thermal comfort studies and ENVI-met simulations. In this equation, *T*_*g*_ denotes globe temperature (°C), *V*_*a*_ is the wind speed (m/s), *D* represents globe diameter (75 mm in this study), *ε* is the globe’s emissivity (0.95 for a black globe), and *T*_*a*_ refers to air temperature (°C).

The experimental setup is illustrated in Fig. 1, and the corresponding conditions are detailed in Table 2.

**Table 2:** Parameters related to the outdoor monitoring equipment.

Variable	Equipment	Measurement range	Accuracy
Air temperature (*T*_*a*_)	PLC-16025	− 40–80 °C	± 0.2 °C
Relative humidity (RH)	PLC-16025	0–100%	± 2%
Wind speed (*V*_*a*_)	PLC-16025	0–15 m/s	± 0.3 m/s
Wind direction (WD)	PLC-16025	0–360°	± 3°
Black sphere temperature (*T*_*g*_)	AZ8778 with a radius of 75 mm	0–50 °C	± 0.61 °C

#### Validating the experimental model reliability

Linear regression analysis was employed to compare the simulation outputs with field measurements. The meteorological variables used for model validation included air temperature (*T*_*a*_), mean radiant temperature (*T*_*mrt*_), and physiologically equivalent temperature (PET). Model performance was evaluated using the coefficient of determination (R^2^), root mean square error (RMSE), and mean absolute error (MAE). RMSE represents the square root of the average squared differences between the simulated and observed values, while MAE reflects the average magnitude of the prediction errors. The equations for RMSE and MAE are as follows:23

Where *S*_*i*_ denotes the simulated values, *M*_*i*_ represents the measured value, and *n* represents the total number of data points (*n* = 36).

**Fig. 5:**
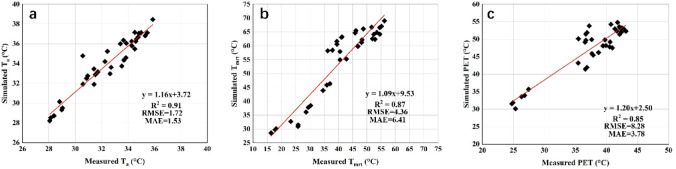
Comparison between simulated and observed values for three microclimatic indicators: **a** air temperature (*T*_*a*_), **b** mean radiant temperature (*T*_*mrt*_), and **c** Physiological Equivalent Temperature (PET).

Model validation was conducted using three statistical metrics: the coefficient of determination (R^2^), mean absolute error (MAE), and root mean square error (RMSE). R^2^ quantifies the strength of the correlation between observed and simulated values, with values approaching 1 indicating higher model reliability. The R^2^ values for air temperature (*T*_*a*_), mean radiant temperature (*T*_*mrt*_), and physiologically equivalent temperature (PET) were 0.91, 0.895 and 0.848, respectively (Fig. 5). Corresponding MAE values were 1.53 °C for *T*_*a*_, 6.41 °C for *T*_*mrt*_, and 3.78 °C for PET, while RMSE values were 1.72 °C for *T*_*a*_, 4.36 °C for *T*_*mrt*_, and 8.28 °C for PET. The ENVI-met simulation used the default solar radiation dataset within its simple forcing model. According to the review by Lam et al.^29^, the obtained R^2^, RMSE and MAE values fall within acceptable performance thresholds for urban microclimate simulations. The strong agreement between simulated and observed value confirms the reliability and applicability of the ENVI-met model in this study.

### Selection of thermal comfort index

This study selected PET as the primary metric for evaluating thermal comfort. PET is a thermal comfort index derived from the Munich Energy-balance Model for Individuals (MEMI) and indicates the equivalent air temperature at which a person in a standard indoor environment would maintain the same physiological conditions, specifically skin temperature and sweat rate, as under the actual outdoor conditions being assessed. Compared to Predicted Mean Vote (PMV) index, PET offer greater accessibility and interpretability, particularly for non-specialists, as it is expressed in degrees Celsius. In addition, PET accounts for a comprehensive set of environmental variables, including air temperature, humidity, wind speed and mean radiant temperature, which enhances its physiological relevance for evaluating outdoor thermal comfort. Owing to its basis in human energy balance, PET more accurately captures thermal stress and subjective comfort in diverse environmental contexts. Given these advantages, PET was selected as the thermal comfort evaluation index in this study. It is calculated using the following heat balance equation:4

Where *S* is the rate of heat storage in the body, *M* is metabolic heat production, *W* is external mechanical work, *R* is net radiation exchange, *C* is convective heat flow, *K* is conductive heat exchange, *E* is evaporative heat loss, and *RES* represents respiratory heat exchange (including both latent and sensible components).

### Design of improvement schemes

**Fig. 6:**
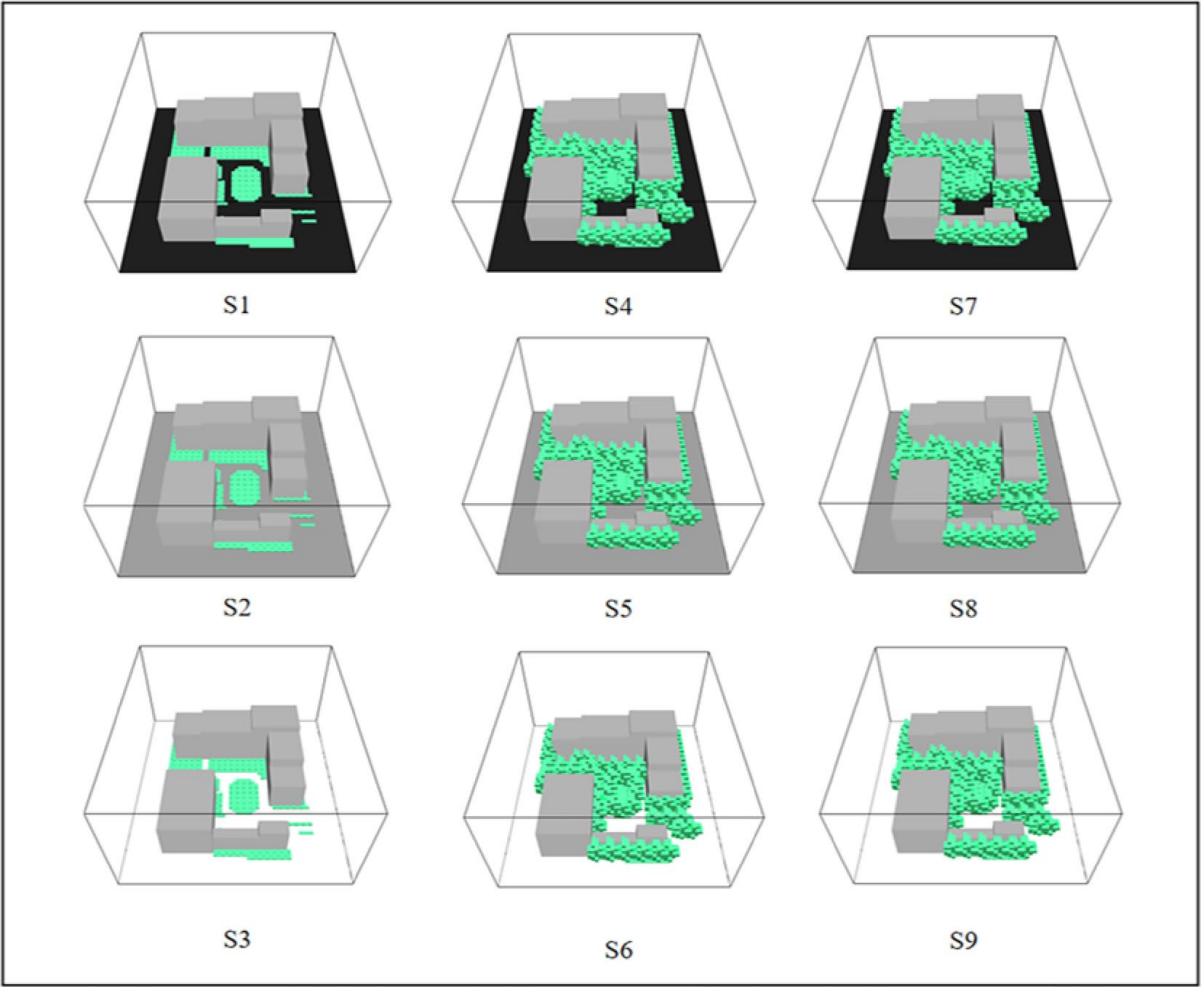
ENVI-met models of the nine simulated scenarios. S1: “Lawn + Shrubs + Asphalt paving”. S2: “Lawn + Shrubs + Concrete paving”. S3: “Lawn + Shrubs + High-reflective concrete paving”. S4: “Lawn + Trees + Asphalt paving”. S5: “Lawn + Trees + Concrete paving”. S6: “Lawn + Trees + High-reflective concrete paving”. S7: “Lawn + Shrubs + Trees + Asphalt paving”. S8: “Lawn + Shrubs + Trees + Concrete paving”. S9: “Lawn + Shrubs + Trees + High-reflective concrete paving”.

ENVI-met software was used to simulate and improve the summer microclimate within the study area. Vegetation and ground paving materials were treated as the primary variables, while all other parameters were held constant. The simulation was conducted over a 12-h period, from 07:00 to 18:00, to capture key diurnal microclimatic variations. Temporal changes in relative humidity (RH), wind speed (*V*_*a*_), air temperature (*T*_*a*_), and surface temperature (*T*_*s*_) were analyzed to evaluate the interactive effects of different vegetation and pavement configurations. Figure 3 illustrates the base ENVI-met model, including the spatial extent and building geometry of the study site. A total of nine representative design combinations were simulated (Fig. 6), and the corresponding spatial distributions of *T*_*a*_, *T*_*s*_, RH, and PET were examined to assess their microclimatic impacts.

### Data analysis

Air temperature (*T*_*a*_), relative humidity (RH), surface temperature (*T*_*s*_), and physiological equivalent temperature (PET) were analyzed at a height of 1.4 m in a campus courtyard across nine intervention scenarios and one baseline condition to assess the combined effects of different design strategies on thermal conditions and comfort. To quantify the thermal performance of each scenario, the mean values of microclimatic parameters and thermal indices were calculated. Differences between each scenario and the baseline were then determined using the following expression:5

Where *ΔS*_*i*_ denotes the difference between scenario *i* and the baseline scenario, *S*_*i*_ represents mean value of scenario *i*, and *S*_*0*_ represents mean value of the baseline scenario.

## Results

### Effects of paving materials and vegetation on air temperature

**Fig. 7:**
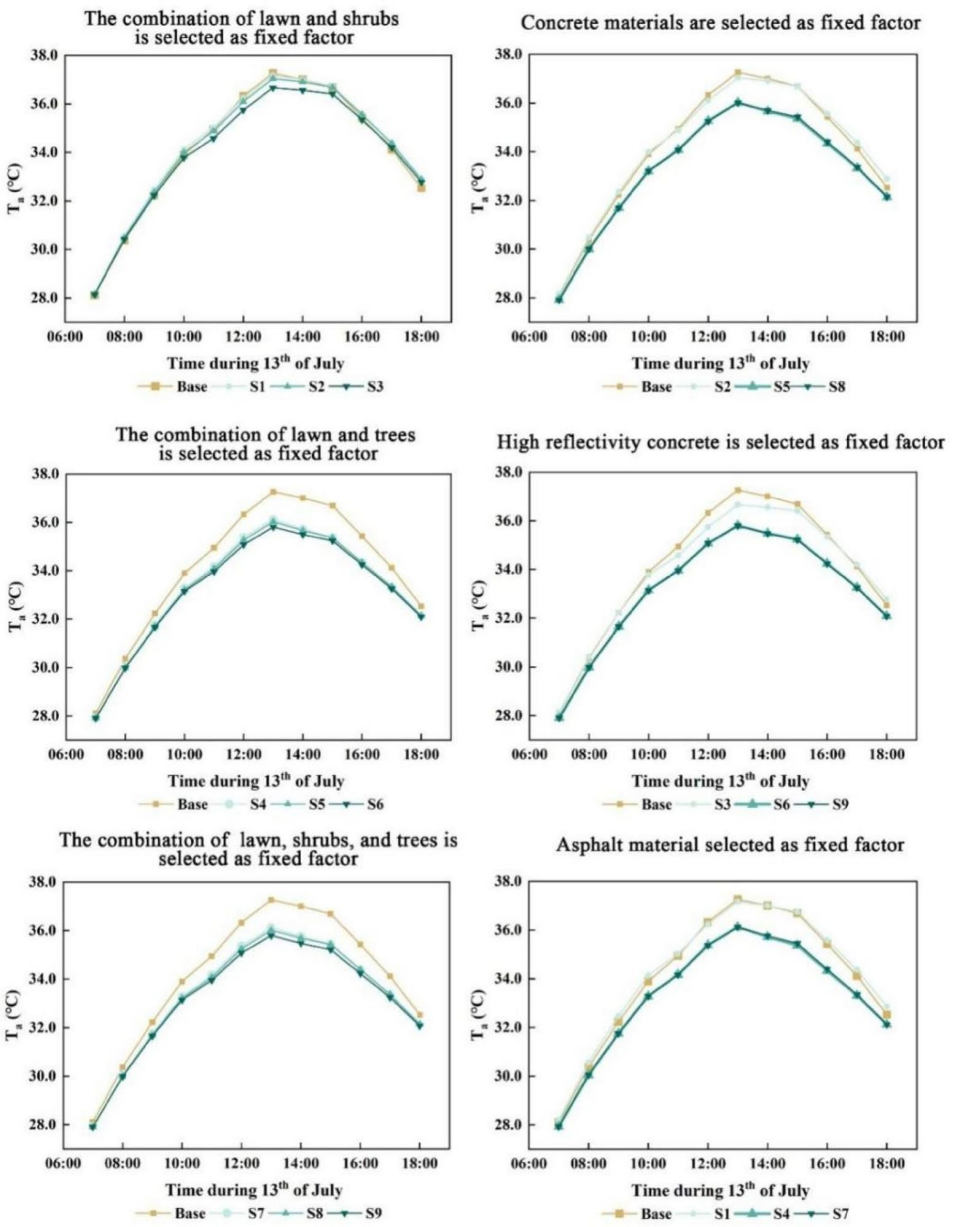
Coupled effects of diverse paving materials and vegetation on air temperature (*T*_*a*_).

**Fig. 8:**
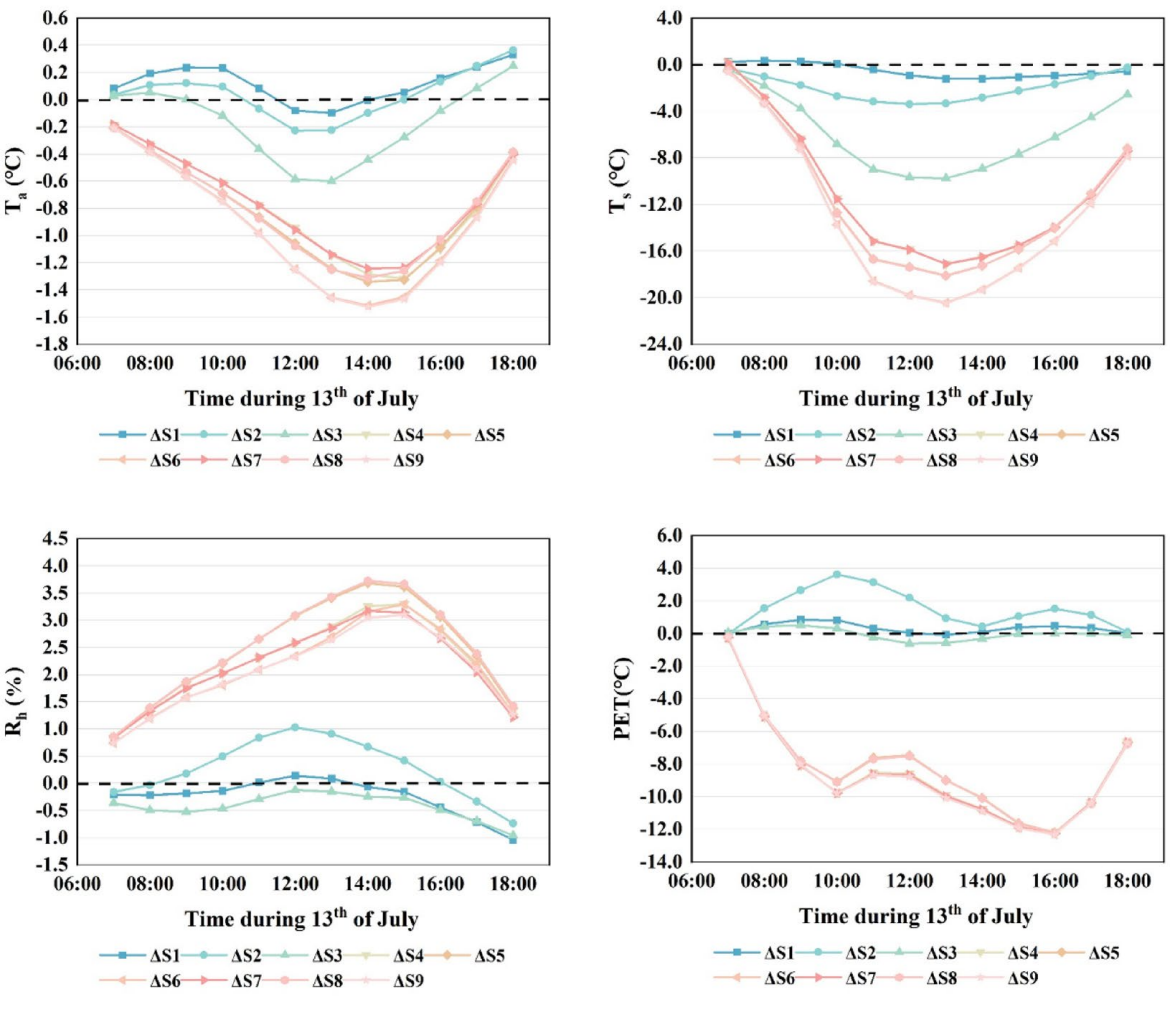
Comparison of air temperature, surface temperature, relative humidity and PET across different scenarios.

The analysis of the combined effects of different paving materials and vegetation types on air temperature (*T*_*a*_) (Figs. 7, 8) revealed a consistent pattern across the simulated scenarios. Air temperature exhibited synchronized variations in response to changes in surface material and vegetation configuration, indicating that these design elements exert a coupled influence on microclimatic conditions.

1. Among the nine simulated scenarios, when vegetation configuration was held constant, the use of concrete with a reflectivity of 0.5 demonstrated notable cooling effects compared to the baseline and other paving materials. Under the “lawn + shrubs” setting, it reduced air temperature notably between 12:00 and 15:00, reaching 36.48 °C at 13:00, 0.63 °C lower than the baseline. Under the “lawn + trees” configuration, the same material achieved the greatest overall cooling from 09:00 to 18:00, with a temperature of 35.63 °C at 14:00, corresponding to a 1.52 °C reduction relative to the baseline. A similar effect was observed in the “lawn + shrubs + trees” scenario, with a temperature of 35.62 °C at 14:00, a 1.53 °C below the baseline. These results indicate that high-reflectivity concrete is most effective when combined with tree-based vegetation structures.
2. When paving material was held constant, vegetation configuration significantly influenced cooling performance. With concrete, the “lawn + shrub” combination produced minimal temperature reduction, while both “lawn + tree” and “lawn + shrub + tree” configurations yielded comparable and substantial cooling between 08:00 and 18:00, peaking at 14:00 (Fig. 7). A similar pattern was observed with asphalt, though the overall cooling effect was weaker. When high-reflectivity concrete (reflectivity = 0.5) was applied, all three vegetation configurations, including “lawn + shrub”, “lawn + tree” and “lawn + shrub + tree”, produced significant reductions in air temperature. The most pronounced effects were again observed in the “lawn + tree” and “lawn + shrub + tree” scenarios, with both reaching 35.62 °C at 14:00, representing a 1.53 °C decrease from the baseline. These findings highlight the critical role of tree vegetation, particularly when paired with reflective paving, in enhancing microclimatic cooling.
3. The simulation results indicate that increasing the reflectivity of paving materials could significantly reduce air temperature, and tree vegetation also had a significant effect on reducing air temperature. In contrast, shrub vegetation has minimal impact, and lawn cover provides the least cooling. Thus, trees and high-reflectivity paving materials are the primary factors influencing air temperature.

### Effects of paving materials and vegetation on relative humidity

**Fig. 9:**
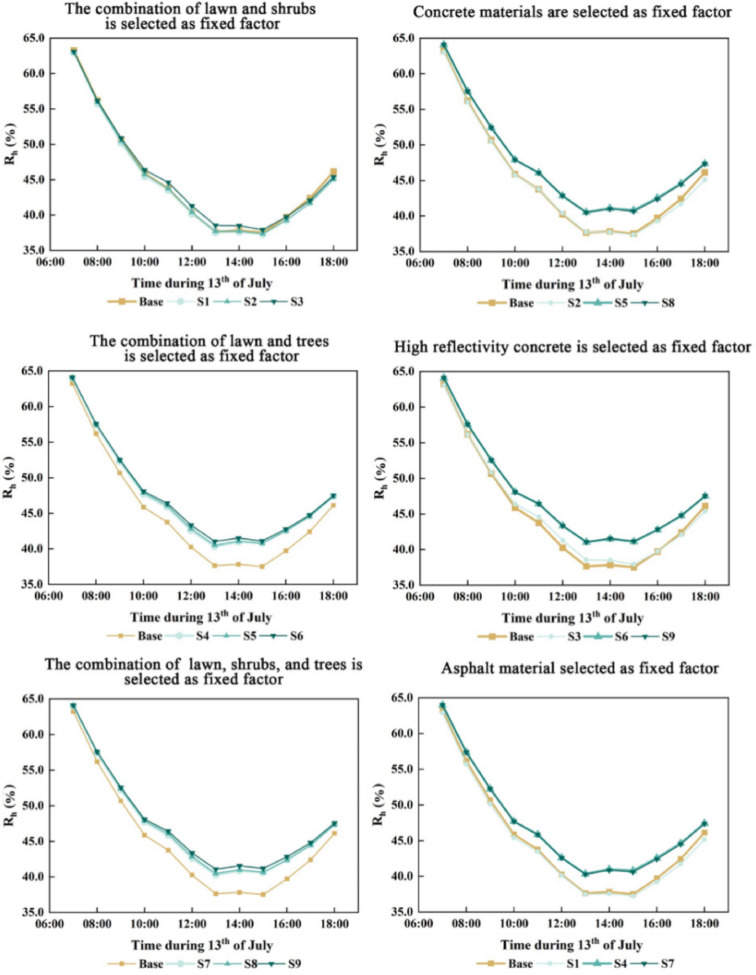
Coupled effects of diverse paving materials and vegetation on relative humidity (RH).

The coupled effects of different paving materials and vegetation on relative humidity were analyzed, revealing a synchronized pattern of variation in the simulated relative humidity values (Figs. 8, 9).

1. Among the nine coupling scenarios, with vegetation held constant, the “lawn + shrub” combination showed no significant difference in relative humidity when paved with concrete or asphalt compared to the baseline. However, using 0.5 reflectivity concrete led to a slight increase in relative humidity from 09:00 to 16:00, peaking at 1% above baseline at 12:00 (Fig. 9). For the “lawn + tree” configuration, all three paving materials increased relative humidity significantly, with 0.5 reflectivity concrete producing the highest peak of 3.69% at 14:00. The “lawn + shrub + tree” combination exhibited similar trends, with the 0.5 reflectivity concrete scenario reaching a slightly lower peak increase at 14:00 compared to the “lawn + tree” scenario.
2. When paving material was held constant and concrete was selected, relative humidity in the “lawn + shrub” scenario remained comparable to the baseline. In contrast, both the “lawn + tree” and “lawn + shrub + tree” scenarios exhibited significant increases, peaking at 14:00 with rises of 3.3% and 3.2%, respectively. Asphalt produced similar trends, with the “lawn + tree” combination showing a slightly higher increase than “lawn + shrub + tree”. Under 0.5 reflectivity concrete, all three vegetation configurations showed elevated relative humidity, with the “lawn + shrub + tree” combination exceeding the “lawn + tree” by 0.03% on average. This may result from lower surface and air temperatures under high-reflectivity concrete, enhancing shrub interaction and thus promoting humidity increases.
3. The simulation results indicate that among the vegetation types, trees had the strongest positive influence on relative humidity, whereas shrubs slightly reduced it. Paving materials had a minor effect, with concrete increasing relative humidity more than asphalt. This effect was further enhanced by increasing the concrete’s reflectivity.

### Effects of paving materials and vegetation on surface temperature

**Fig. 10:**
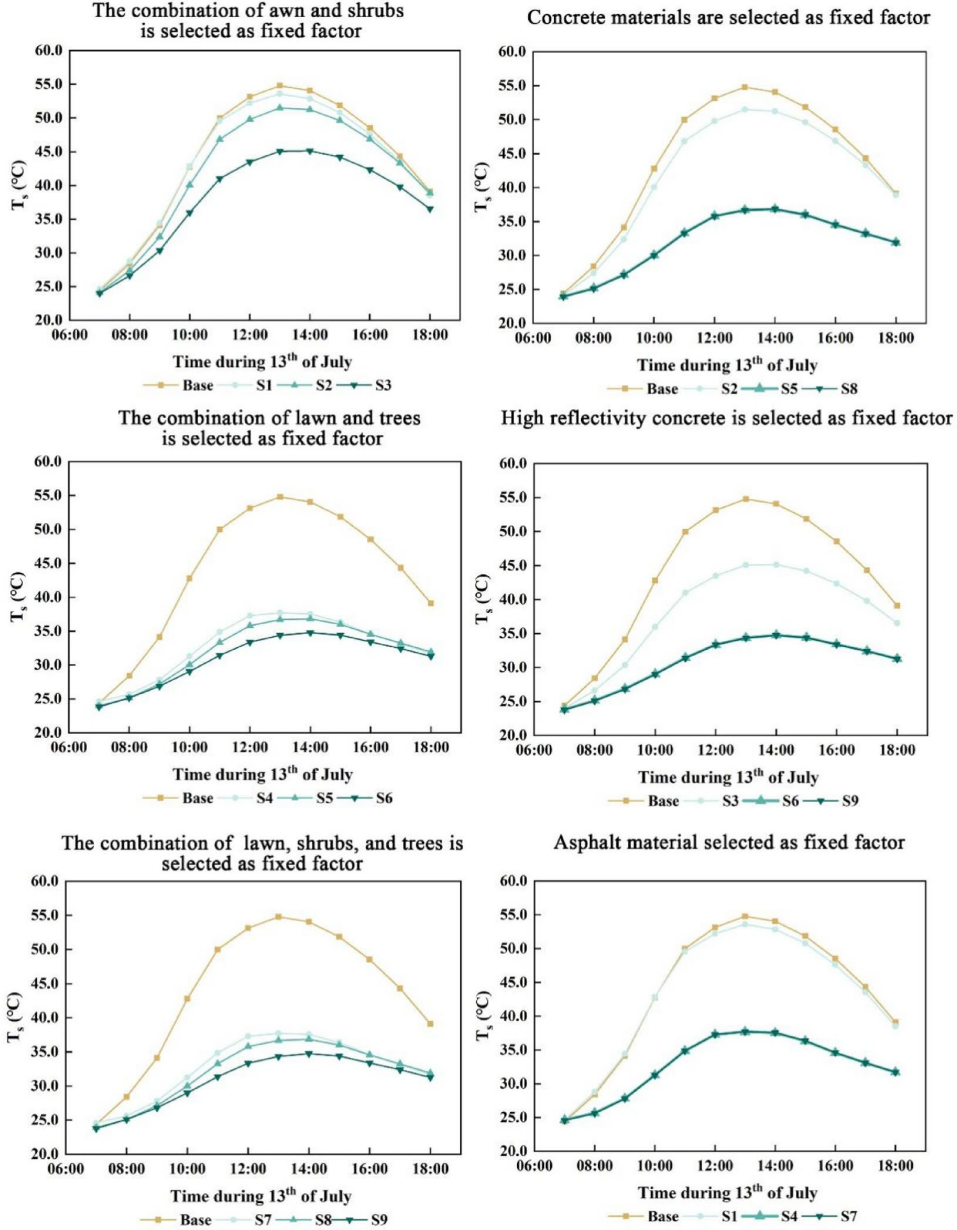
Coupled effects of diverse paving materials and vegetation on surface temperature (*T*_*s*_).

The analysis of various combinations of paving materials and vegetation revealed a consistent pattern of surface temperature variation across the nine simulated scenarios (Figs. 8, 10), indicating synchronized responses to the coupled design interventions.

1. When vegetation was held constant and the “lawn + shrub” configuration was applied, concrete with a reflectivity of 0.5 significantly reduced surface temperature, reaching a peak reduction of 9.79 °C at 13:00 compared to the baseline. Standard concrete also yielded a cooling effect, though markedly less pronounced. In contrast, asphalt provided minimal mitigation and resulted in higher surface temperatures than the baseline between 11:00 and 18:00. Under the “lawn + tree” configuration, all three paving materials produced substantial cooling. High-reflectivity concrete resulted in the greatest temperature reduction, with a decrease of 20.57 °C at 13:00, while standard concrete and asphalt led to reductions of 18.25 °C and 17.18 °C, respectively. The “lawn + shrub + tree” configuration exhibited a similar cooling pattern to “lawn + tree”, with a marginally greater overall cooling effect.
2. When paving material was held constant and concrete was selected, the “lawn + shrub” combination produced a modest cooling effect, particularly between 9:00 and 16:00. In contrast, the “lawn + tree + concrete” and “lawn + shrub + tree + concrete” configurations exhibited pronounced cooling, both peaking at 13:00 with reductions of 18.25 °C and 18.28 °C, respectively–the latter performing slightly better. When asphalt was used, cooling was generally weaker across all combinations, with the “lawn + shrub” configuration showing the greatest decline in cooling effectiveness. Substituting concrete with a high-reflectivity variant (reflectivity = 0.5) markedly enhanced the cooling effect of the “lawn + shrub” configuration and slightly improved that of the “lawn + tree” and “lawn + shrub + tree” combinations. These latter two reached peak cooling at 13:00, with temperature reductions of 20.57 °C and 20.61 °C, respectively, with the latter offering a marginally greater benefit.
3. Simulation results identified paving material reflectivity and tree presence as the primary factors influencing surface temperature. High-reflectivity concrete (reflectivity = 0.5) produced the greatest cooling effect, followed by standard concrete, while asphalt was least effective. Thus, enhancing pavement reflectivity and incorporating trees into vegetation design can substantially reduce surface temperature.

### Effects of paving materials and vegetation on human thermal comfort

The PET values for the nine coupling scenarios were calculated using the Bio-met module of ENVI-met software. Since variations in mean radiant temperature directly impact human thermal comfort, this study focused on these simulated results. The analysis revealed that the human thermal comfort outcomes across the nine coupling types exhibited a consistent, synchronized trend.

**Fig. 11:**
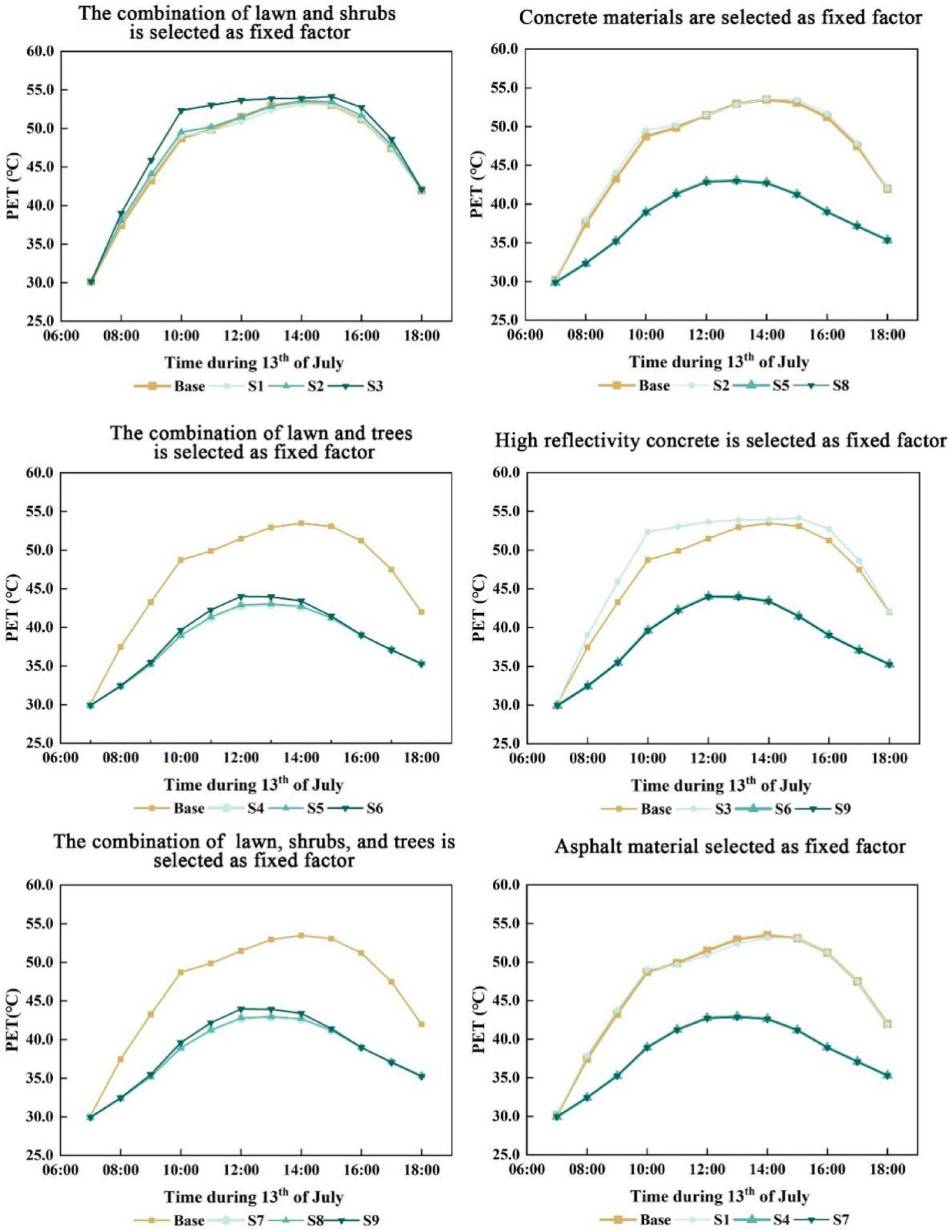
Coupled effects of diverse paving materials and vegetation on human thermal comfort (PET).

1. With vegetation held constant, the “lawn + shrub” configuration yielded comparable thermal performance for concrete and asphalt pavements (Fig. 11). Concrete with 0.5 reflectivity consistently showed poorer thermal comfort, especially between 09:00–13:00 and at 16:00. Peak PET values at 14:00 were 53.94 °C for high-reflectivity concrete, 53.60 °C for standard concrete, and 53.37 °C for asphalt, all exceeding baseline levels. The high-reflectivity concrete peaked earlier at 11:00, indicating a faster surface warming. Under the “lawn + tree” configuration, all three paving materials significantly improved thermal comfort, with PET peaks at 13:00 of 44.49 °C, 43.32 °C, and 43.18 °C for high-reflectivity concrete, standard concrete, and asphalt, respectively. These results indicate that standard concrete and asphalt provided better thermal comfort than high-reflectivity concrete. The “lawn + shrub + tree” configuration showed similar trends, with only marginal further PET reduction, suggesting limited additional benefit (Fig. 8).
2. With paving material held constant, the “lawn + shrub” configuration using concrete yielded results largely consistent with the baseline. In contrast, the “lawn + tree” and “lawn + shrub + tree” combinations significantly enhanced thermal performance, producing nearly identical improvements. When asphalt was used, PET trends across vegetation types mirrored those with concrete, but overall thermal comfort gains were slightly greater. Conversely, applying high-reflectivity concrete (reflectivity 0.5) reduced the thermal performance of the “lawn + shrub” configuration and slightly diminished the effectiveness of the “lawn + tree” and “lawn + shrub + tree” combinations compared to standard concrete or asphalt. Nonetheless, the general pattern of thermal comfort improvement across configurations remained consistent.
3. Simulation results indicate that tree planting markedly improved courtyard thermal comfort by significantly reducing PET, identifying it as the most influential factor. Shrubs contributed only modest reductions. The type of paving material had a comparatively limited impact; however, high-reflectivity surfaces such as concrete with a reflectivity of 0.5, increased PET due to increased mean radiant temperature (*T*_*mrt*_), despite lowering air temperature (*T*_*a*_) and surface temperature (*T*_*s*_). For instance, in scenario S6, *T*_*a*_ at 14:00 was 35.63 °C, and *T*_*s*_ decreased by 20.57 °C at 13:00, attributed to enhanced solar reflectance. However, this also intensified shortwave radiation, raising *T*_*mrt*_. In scenario S3, *T*_*mrt*_ at 14:00 was approximately 6 °C higher than the baseline.

## Discussion

In recent decades, the rate of global temperature increase has accelerated markedly^30^, a trend projected to persist, with adverse implications for the thermal comfort of outdoor spaces in urban environments^10^. Accurately assessing thermal comfort in urban green spaces remains a major challenge for effective urban planning, due to the complex configuration of these spaces and the fact that thermal comfort is a multidimensional perception shaped by both physiological and psychological factors. Among the available predictive tools, the ENVI-met model is one of the most widely applied across diverse climatic contexts and urban morphologies, owing to its capacity to simulate complex interactions among buildings, vegetation, and microclimate. Recent literature has confirmed that ENVI-met offers reasonably accurate estimations of key parameters such as air temperature (*T*_*a*_) and mean radiant temperature (*T*_*mrt*_)^11,31^. However, thermal comfort in any given green space is influenced not only by *T*_*a*_ and *T*_*mrt*_, but also by a range of micro-meteorological variables. As such, simulation results should be interpreted as idealized representations, capturing general thermal trends rather than precise real-world conditions.

### Vegetation and paving impact thermal comfort

Simulation results revealed that both vegetation and paving materials significantly influence thermal comfort in the study area. Appropriate configuration of vegetation and paving materials can significantly improve the thermal environment and enhance outdoor comfort. Among all influencing factors, air temperature (T_*a*_) plays a dominant role, as increased *T*_*a*_ amplifies surface radiation and intensifies both thermal discomfort and microclimatic stress.

Vegetation type played a key role in regulating air temperature. Tree-covered configurations markedly reduced *T*_*a*_ due to their shading capacity, which limits direct solar radiation and surface heating^23^. In contrast, shrubs provided limited shade and were less effective in moderating *T*_*a*_, allowing rapid increases in surface and air temperatures.

Paving materials also contributed to thermal performance. Asphalt, with its high heat capacity and low albedo, retained more heat and produced higher *T*_*a*_ compared to concrete and highly reflective concrete. In contrast, highly reflective concrete, with its light-colored surface, low heat storage, and strong solar reflectance, significantly reduced air temperatures, thereby enhancing thermal comfort^32,33^. Surface temperature (*T*_*s*_), closely linked to the urban heat island effect^34^, was significantly reduced in configurations combining tree shade and lawn evapotranspiration^23^. While shrubs also offered some cooling, their effect was notably weaker than that of trees. The combination of trees, shrubs, and lawn yielded the greatest *T*_*s*_ reduction.

Surface temperature trends varied significantly by paving material. Asphalt surfaces exhibited the highest midday *T*_*s*_, followed by standard concrete, while highly reflective concrete achieved the lowest surface temperatures. Combinations with higher green coverage (e.g., “lawn + trees” and “lawn + shrubs + trees”) also maintained higher relative humidity during simulations. This was primarily due to increased evapotranspiration, which elevated ambient moisture levels^35^. Reflective paving materials indirectly contributed to higher humidity by lowering *T*_*s*_ and thereby suppressing evaporative losses^36^.

### Trees enhance thermal comfort via shading and evapotranspiration

Trees demonstrated the greatest improvement in thermal comfort, as reflected in PET, through combined shading and evaporative cooling. In contrast, shrubs contributed only marginal improvements. Although highly reflective concrete effectively reduced *T*_*s*_ and *T*_*a*_, it also increased *T*_*mrt*_ under direct solar exposure, potentially offsetting its cooling advantages. Previous studies have shown that high reflectivity surfaces can increase *T*_*mrt*_ by 3–5 °C^37^, diminishing the net thermal benefit of reduced *T*_*a.*_ Given the high sensitivity of PET to *T*_*mrt*_^10^, scenario S6 reached a PET of 44.49 °C, exceeding the 43.32 °C recorded in S5. In the absence of shading, high-reflectivity surfaces can elevate *T*_*mrt*_ by up to 7 °C^38^, leading to extreme PET values (e.g., 53.94 °C in scenario S3), which substantially reduce outdoor usability. However, tree canopy coverage, as in scenario S6, mitigated this effect by limiting *T*_*mrt*_ increases to approximately 3 °C, thereby contributing to meaningful PET reductions.

### Practical considerations

These findings highlight the necessity of combining high-reflectivity materials with sufficient tree cover to improve thermal comfort. At the campus scale, additional factors such as maintenance, cost, and ecological value must be considered. Vegetation, particularly in hot and humid climates like Hengyang, demands ongoing care, including irrigation and pest management^14^. In contrast, reflective concrete requires minimal maintenance but periodic cleaning to maintain its albedo. Initial costs vary considerably: tree planting ranges from $50 to $100 per tree, lawns cost $2 to $5 per square meter, and reflective concrete ranges from $10 to $15 per square meter, exceeding the $5 to $10 per square meter cost of standard concrete^39^. Furthermore, monoculture plantings simplify maintenance but diminish biodiversity, whereas mixed-species vegetation enhances ecological benefits while increasing management complexity^6^. Effective campus planning should therefore balance thermal comfort, cost efficiency, and biodiversity according to the functional requirements of each area.

### Microclimate optimization strategies

The findings demonstrate that a scientifically informed configuration of green spaces and paving materials can effectively mitigate the urban heat island effect, improve local microclimates, and enhance residents’ thermal comfort. Specifically, combining trees and lawns substantially reduced air and surface temperatures, making this approach a priority for densely populated residential areas, public spaces, and schools where outdoor comfort is essential. Conversely, in high-traffic corridors and commercial zones requiring greater durability and load-bearing capacity, highly reflective concrete should be employed to minimize surface heat buildup near sidewalks and buildings. Extensive use of asphalt in major activity areas is discouraged due to its high thermal capacity, which exacerbates overheating. These insights provide a basis for urban planners to establish green space ratio standards and optimize tree and shrub configurations, ensuring diverse vegetation and paving materials. This integrated strategy aims to deliver comprehensive cooling and improve local thermal comfort.

To enhance the applicability of these results, future research will conduct cross-climate and cross-regional analyses to address existing limitations. Since the cooling effects of vegetation are climate-dependent^6^, the “trees + lawn” model will be tested in diverse environmental contexts–including simulations in Beijing (cold, dry) and Guangzhou (tropical)–to assess its generalizability. The study will also expand to additional campus settings, such as sports fields and dormitories, in order to develop tailored microclimate design strategies. Moreover, innovative materials such as low-reflectivity permeable concrete and heat-tolerant native tree species (e.g., *Cinnamomum camphora*) will be evaluated for their potential to improve both thermal comfort and ecological performance. Consideration of seasonal variation and projected climate change impacts will further inform adaptive, climate-resilient urban planning. This research supports the broader objective of fostering harmonious and sustainable urban environments, thereby enhancing quality of life in the face of ongoing environmental change.

## Limitations and future outlook

This study presents several limitations that highlight key directions for future research. First, the analytical framework employed in this study is based on a predefined set of variables and scenarios designed to identify favorable configuration, thus, the outcomes should be regarded as context-specific evaluations rather than globally optimized solutions derived from formal mathematical or computational models. Second, while the nine coupled scenarios reflected realistic micro-environmental conditions, additional simulations are needed to more comprehensively explore the interdependencies among key microclimatic variables. Third, the analysis was confined to a semi-enclosed campus courtyard, with outcomes shaped by localized architectural features such as building height and orientation; expanding the study to include a broader range of urban typologies would enhance the generalizability of the findings. Finally, the study considered only summer daytime conditions, omitting nighttime and winter scenarios. Future investigations should incorporate full diurnal and seasonal cycles to assess the year-round effectiveness of thermal comfort strategies. Collectively, these limitations highlight the need for more extensive, multi-scalar, and temporally inclusive research to generate robust and transferable insights into urban microclimate regulation and thermal comfort optimization.

## Conclusions

This study examined the summer microclimate of the central teaching area of a university campus in Hengyang using ENVI-met simulation. High-temperature summer days were selected for analysis, and model accuracy was validated through field measurements. Microclimatic responses were assessed across nine vegetation-paving material coupling scenarios. The main conclusions are as follows:

1. Vegetation configuration significantly influenced the microclimate. Scenarios incorporating trees (“lawn + trees” and “lawn + shrubs + trees”) were most effective at reducing air temperature (*T*_*a*_) and surface temperature (*T*_*s*_), increasing relative humidity (RH), and lowering Physiological Equivalent Temperature (PET). In contrast, the “lawn + shrubs” combination had a comparatively weaker effect.
2. Paving material type also affected thermal conditions. High-reflectivity concrete achieved the greatest reductions in *T*_*a*_ and *T*_*s*_, followed by standard concrete and asphalt. However, its elevated reflectivity increased mean radiant temperature (Tmrt), resulting in higher PET and potentially reduced thermal comfort.
3. ENVI-met proved reliable for simulating microclimatic effects of vegetation-paving interactions. Simulated outcomes closely aligned with field measurements, supporting the model’s applicability in optimizing urban thermal environments. The framework presented here offers a basis for developing evidence-based strategies to improve outdoor thermal comfort through landscape and material interventions.

## Data Availability

The datasets used and/or analyzed during the current study are available from the corresponding author Zhihuan Huang on reasonable request via e-mail zhihuan_huang@whu.edu.cn.
